# Imaging Mass Spectrometry and Genome Mining Reveal Antimicrobial Peptides of Novel *Pediococcus acidilactici* CCFM18

**DOI:** 10.3390/foods13142213

**Published:** 2024-07-13

**Authors:** Yiteng Qiao, Fengwei Tian, Leilei Yu, Jianxin Zhao, Qixiao Zhai, Wei Chen

**Affiliations:** 1 College of Food Science and Engineering, Shandong Agricultural University, Tai’an 271018, China; yitengqiao@sdau.edu.cn; 2 State Key Laboratory of Food Science and Resources, Jiangnan University, Wuxi 214122, China; fwtian@jiangnan.edu.cn (F.T.); edyulei@126.com (L.Y.); zhaojianxin@jiangnan.edu.cn (J.Z.); zhaiqixiao@sina.com (Q.Z.); 3 School of Food Science and Technology, Jiangnan University, Wuxi 214122, China; 4 National Engineering Research Center for Functional Food, Jiangnan University, Wuxi 214122, China

**Keywords:** lactic acid bacteria, antimicrobial activity, bacteriocin, imaging mass spectrometry, microbial interactions

## Abstract

The mechanism of metabolites produced by lactic acid bacteria in mediating microbial interactions has been difficult to ascertain. This study comparatively evaluated the antimicrobial effect of the novel bacterium *Pediococcus acidilactici* CCFM18 and explored the global chemical view of its interactions with indicator bacteria. *P. acidilactici* CCFM18 had sufficiently strong antimicrobial activity to effectively inhibit the growth of the indicator bacteria and enhance their intracellular reactive oxygen species (ROS) level. The emerging technique of matrix-assisted laser desorption ionization–time-of-flight (MALDI-TOF) imaging mass spectrometry indicated that *P. acidilactici* CCFM18 increased the production of pediocin PA-1 and the penocin A profile during its interaction with the indicator bacteria, thus differing from *P. acidilactici* CCFM28 (a commonly used laboratory strain). Strikingly, the production of coagulin A was triggered only by signaling molecules made by the competing strain *L. thermophilus*, suggesting an idiosyncratic response from *P. acidilactici* CCFM18. Bioinformatic mining of the *P. acidilactici* CCFM18 draft genome sequence revealed gene loci that code for the complex secondary metabolites analyzed via MSI. Taken together, these results illustrate that chemical interactions between *P. acidilactici* CCFM18 and indicator bacteria exhibit high complexity and specificity and can drive *P. acidilactici* CCFM18 to produce different secondary metabolites.

## 1. Introduction

Public awareness of the environmental and economic concerns associated with food waste has recently been raised [1]. Some spoilage and pathogenic microorganisms easily contaminate food and deteriorate food items during storage by producing health-hazardous toxic secondary metabolites [2]. The associated risk factors in humans are related to the consumption of contaminated food and water, as well as direct contact with contaminated food [3]. Furthermore, the treatment of wasted food is a great environmental challenge. Thus, it is vitally important to find a food preservation strategy to avoid contamination due to spoilage and pathogenic microorganisms during food storage [4]. During recent years, as a green technology, biopreservation—which extends the shelf life and enhances the safety of foods by using microorganisms or their antimicrobial metabolites—has attracted attention [4].

Beneficial microorganisms responsible for the suppression of pathogen growth have been discovered among bacteria of several genera, e.g., *Bacillus*, *Alcaligenes*, *Burkholderia*, *Agrobacterium*, and *Pseudomonas* [5,6]. Of these, lactic acid bacteria (LAB) are receiving much attention. In applications as ‘natural’ biopreservatives, some strains of LAB produce bacteriocins, as well as lactic and acetic acids, propionic acids, sorbic acids, benzoic acids, hydrogen peroxide, and phenolic and proteinaceous compounds that have antibacterial functions [5]. In addition, some LAB can inhibit enteric pathogens via the generation of reactive oxygen species (ROS), such as H_2_O_2_ and NO_x_ species, which are considered to have a long lifetime and high bacteriostatic efficiency [1]. A lactic acid bacterium strain was previously isolated from a pickle in Wuxi, China [7]. This bacterium, named *P. acidilactici* CCFM18, inhibits the pathogenic bacterium *L. monocytogenes* in vitro and functions across a large range of pH and temperature values, showing high activities at temperatures from 60 °C to 121 °C [8]. The properties of *P. acidilactici* CCFM18 show high potential for economic applications; however, previous attempts to identify antimicrobial compounds, such as proteinaceous and bacteriocin compounds, in *P. acidilactici* CCFM18 were unsuccessful.

Several other studies have attempted to identify the antimicrobial products of LAB [4]. Only a few bacteriocins that showed strong inhibitory effects on *Listeria* have been discovered and characterized [4,9]. A number of LAB fight the competing Gram-positive microorganisms by secreting secondary metabolites in the interaction zone [4]. Traditional metabolomic tools allow for the analysis of thousands of metabolites into a homogenate simultaneously; however, the extraction procedure destroys the spatial organization of microbe—microbe interactions, and metabolites may be an essential reason for the previous failure to decipher antimicrobial products [10]. Thus, visualization of the distribution of metabolites is important to understand the molecular interactions between LAB and their competitors [11]. Mass spectrometry imaging (MSI) technology visualizes site-specific chemical molecules in situ and generates the distribution patterns of metabolites, thereby elucidating the bioactive compounds in interactions between microbes in high spatio-temporal resolution [12]. Matrix-assisted laser desorption ionization–time-of-flight imaging mass spectrometry (MALDI-TOF MSI) has recently provided new ways of examining the exchange of secondary metabolites between interacting bacteria in situ [13]. Using MALDI-TOF MSI technology, Dorrestein et al. identified a novel diffusible *Ralstonia solanacearum* lipopeptide, ralsolamycin, that has the governing role in mediating interactions between the soil pathogen *R. solanacearum* and soil-associated fungi using MALDI-TOF MSI technology [14]. A previous study reported the use of MALDI-TOF MSI combined with in silico genome analyses to uncover the spatial distribution patterns of antibiotics induced in *Lysobacter* species during interactions with the fungus *Rhizoctonia solani* and trace the biosynthetic origins of the antibiotic compound, thus revealing the antagonist mechanism [15]. Previous studies have shown that MALDI-TOF MS is a rapid and sensitive detection method for bacteriocin research [14].

In this study, for the first time, we used MALDI-TOF MSI in combination with molecular genomics analysis to unravel the antimicrobial mechanism of the *P. cidilactici* CCFM18 strain, which indicated that LAB interspecies’ interactions can trigger broad, differential production of secreted metabolites by a single LAB. The antibacterial activity of the isolated bacterium *P. acidilactici* CCFM18 was screened against four indicator strains (*L. thermophilus*, *L. delbrueckii*, *L. helveticus*, and *E. faecalis*) because other LAB are likely to be the most prominent competitors of LAB in the ecological niche. The strain *P. acidilactici* CCFM28 was also examined in order to compare its antimicrobial action to that of the isolated *P. acidilactici* CCFM18 strain and to identify the core and distinct metabolites.

## 2. Materials and Methods

### 2.1. Bacterial Strains and Culture Conditions

The LAB strain *P. acidilactici* CCFM18 was originally isolated from pickles made by local dwellers in Wuxi via natural fermentation. *P. acidilactici* CCFM28 and the indicator strains (*L. thermophilus*, *L. delbrueckii*, *L. helveticus*, and *E. faecalis*) were all obtained from the Culture and Information Center of Industrial Microorganisms of China Universities, Jiangnan University (CICIM-CU; Wuxi, China). All strains were grown in MRS (deMan–Rogosa–Sharpe) broth (Hopebio Cooperation, Qingdao, China) at 37 °C for 18–24 h.

### 2.2. Determination of Antimicrobial Activity in Liquid Culture and Agar Well Diffusion Experiments

The antimicrobial activity in liquid culture and agar well diffusion experiments was evaluated based on a previous method with some modifications [8]. *P. acidilactici* CCFM18 and *P. acidilactici* CCFM28 at stationary phase (16 h) were quantified using their OD_600_ values and diluted to ~2 × 10^8^ CFU/mL using fresh MRS medium. The cells were harvested via centrifugation (6000× *g*, 10 min, 4 °C), and the supernatants were then filtered through sterilized 0.22 µm Whatman GF/C filters to remove any remaining cells. MRS medium without *P. acidilactici* served as a control. The antimicrobial potential of the LAB supernatants against various indicator bacteria was investigated to analyze extracellular inhibitors. The filtered supernatants of the LAB cultures were transferred (10%, *v*/*v*) into indicator bacterial cultures that had been grown for 24 h at 37 °C and diluted to ~1 × 10^6^ CFU/mL. Bacterial growth was detected by reading the absorbance at 600 nm. To test the effect of *P. acidilactici* on indicator bacterium cultures, the malondizldehyde (MDA) and superoxide dismutase (SOD) contents of the indicator cells in the *P. acidilactici* fermentation liquid were tested every day during incubation. All assays were replicated three times.

An agar well diffusion experiment was also conducted to determine the antimicrobial activity upon LAB evaluation. MRS agar (20 mL) was inoculated with 200 μL of a 24 h cultured indicator organism and poured into a Petri dish. When the agar had set, wells (6 mm in diameter) were punched and filled with 100 μL of supernatants. The plates were maintained at 4 °C for approximately 4 h to aid radial diffusion and then incubated at 37 °C for 48 h. Agar well diffusion tests were performed in three independent experiments, and the average inhibition zones around the wells containing LAB were recorded and calculated.

### 2.3. Detection of the ROS Level

A high ROS level induces oxidative injury in bacteria and serves as an indicator of oxidative stress [16]. Therefore, to study whether antimicrobial activity could induce oxidative stress in the indicator bacteria, the intracellular ROS levels in bacteria incubated with LAB supernatant were measured using the peroxide-sensitive fluorescent probe H_2_DCFDA (2′,7′-dichlorodihydrofluorescein diacetate, Aladdin, Shanghai, China), according to a previously reported method with minor modifications [17]. In brief, indicator bacteria before and after antimicrobial treatment were collected by means of 5000× *g* for 10 min at 4 °C; then, the cells were washed three times using 0.1 M PBS (pH 7.2) and recovered with the same buffer. Thereafter, 50 μL of cell suspension was reacted in 50 μL of 20 μM H_2_DCFDA at 37 °C for 30 min and washed again using PBS (0.1 M, pH 7.2). The cells were then resuspended in PBS at 37 °C for 15 min and analyzed at 495/525 nm (excitation/emission) using a SpectraMax M2e microplate reader (Molecular Devices, San Jose, CA, USA).

### 2.4. Antioxidant Enzyme Assays

The MDA content was determined spectrophotometrically using the thiobarbituric acid method [18]. The cells were sonicated in an ice bath for 30 min, and the absorbance was analyzed at 600, 532, and 450 nm (denoted as OD_600_, OD_532_, and OD_450_, respectively), measured using a spectrophotometer. The concentration of MDA (C_MDA×nM_) in this work was calculated using Equation (1).

C_MDA×nM_ = (6.45(OD_532_ − OD_600_) − 0.56 × OD_450_) × 1000 (1)

The total enzymatic activities of SOD were determined using Diagnostic Reagent Kits (Nanjing Jiancheng Bioengineering Institute, Nanjing, China) according to a previous method with some modifications [19]. The SOD activity was measured using the xanthine oxidase method according to the generation of oxidized hydroxylamine, which produces a red material after 20 min of reaction at 37 °C [19]. This was analyzed by determining the absorbance at 450 nm.

### 2.5. Pairwise Microbial Competition Assays

Ov ernight cultures of all strains were diluted to 0.1 OD_600_; then, 5 μL of each culture was spotted as spots onto thin MRS agar plates (11 mL) according to the method described in a previous study [14]. In this assay, one loopful of *P. acidilactici* CCFM18 and *P. acidilactici* CCFM28 culture (5 μL) was first dropped onto MRS agar plates and dried for 1 h; then, an indicator bacterium culture (5 μL) was dropped in one direction at a 5 mm distance from the *P. acidilactici* spot. The experiments were performed in triplicate, and the plates were incubated for 24 h at 37 °C. After incubation, the bacterial colonies were checked visually, and the interaction at the interface of the microorganisms was recorded.

### 2.6. MALDI-TOF Imaging Mass Spectrometry

Small pieces of MAR that contained colonies of microorganisms (both single and interacting) were cut with a razor blade and transferred to a MALDI imaging plate to conduct MALDI-TOF MSI. The matrix preparation process was conducted as previously described [20]. In brief, 20 mg/L of a universal matrix (1:1 mixture of 2,5-dihydroxybenzoic acid [DHB] and α–cyano-4-hydroxycinnamic acid [HCCA]; positive ion mode) or 9-aminoacridine (negative ion mode) was deposited onto the plates using an HTX TM-Sprayer (HTX Technologies, Carrboro, NC, USA) according to the following settings: flow rate of 0.1 mL/min at 65 °C, track speed of 800 mm/min, and track spacing of 3 mm. The samples were dried in vacuum at 50 °C and then subjected to MSI measurement. Mass calibration and tuning were performed with a PepMix II standard solution (Bruker Daltonics GmbH, Bremen, Germany) in quadratic mode. Imaging of the samples was performed on a Rapiflex MALDI Tissuetyper™ TOF/TOF MS (Bruker Daltonics GmbH, Bremen, Germany), which was operated in both reflection positive-mode and negative-mode analysis according to the following settings: spatial resolution of 50 μm, laser frequency of 200 Hz, and mass range from *m*/*z* 100 to *m*/*z* 5000. The MS images were viewed using FlexImaging 5.0 and SciLs Lab 2018b software (Bruker Daltonics GmbH, Bremen, Germany). A database search to identify the metabolites was conducted with the Human Metabolome Database, Metlin, Massbank, NIST, and Lipidmaps and with reference to previous studies.

### 2.7. DNA Extraction, Genome Sequencing, and Assembly

A culture of *P. acidilactici* CCFM18 or *P. acidilactici* CCFM28 was grown in MRS for 24 h, the cells were collected via centrifugation (6000× *g*, 10 min, 4 °C), and then the genomic DNA was isolated using the Qiagen 100/G kit. Library preparation and 350-base-paired end sequencing were performed at Novogene Company on an Illumina PE150 system. At least 100-fold coverage was obtained for all genome sequences produced in this work. After low-quality data were removed, clean data for strain *P. acidilactici* CCFM18 or *P. acidilactici* CCFM28 were downloaded from http://www.jcvi.org (accessed on 30 March 2021). Genomes were assembled using SOAP denovo (version 2.04) with default settings. Krskgf (version 1.2) and gapclose (version 1.12) software were used to optimize the assembly results, and fragments of less than 500 bp were filtered out.

### 2.8. Genome Analysis

Functional annotations were conducted against the Clusters of Orthologous Groups (COG) database, which comprises 2091 orthologous groups of proteins [21]. BAGEL is a hidden Markov model (HMM)-based software tool that enables genome mining for bacteriocin and its biosynthetic clusters in a knowledge-based database [22]. The genome sequence of *P. acidilactici* CCFM18 was retrieved from BACTIBASE and NCBI and used as a query for BAGEL4 to identify the putative bacteriocin operons.

### 2.9. Statistical Analysis

All experiments were repeated three times. All analysis data are presented as mean ± standard deviation. The significant differences were analyzed using SPSS (Version 21, SPSS Inc., Chicago, IL, USA) with a one-way analysis of variance (ANOVA) followed by Duncan’s test, where *p* < 0.05 is considered statistically significant.

## 3. Results

### 3.1. Antimicrobial Activity of P. acidilactici

**Figure 1:**
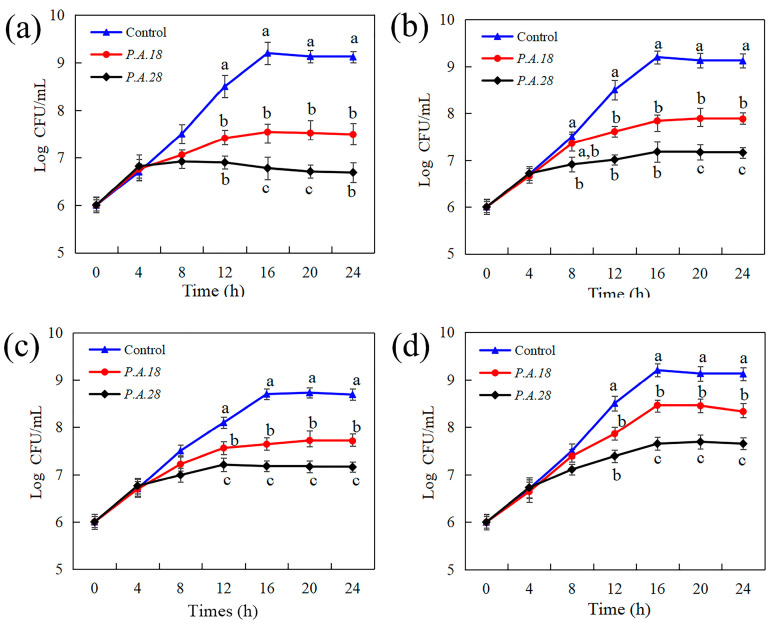
Effect of antimicrobial substances from *P. acidilactici* CCFM18 (*P.A.18*) and *P. acidilactici* CCFM28 (*P.A.28*) supernatants on the cell growth of (**a**) *L. thermophilus*, (**b**) *L. delbrueckii*, (**c**) *L. helveticus*, and (**d**) *E. faecalis* during 24 h of treatment. MRS medium without *P. acidilactici* served as a control. Different letters at the same time indicate statistical differences (*p* < 0.05).

The antibacterial activity of *P. acidilactici* extracts was first investigated against indicator microorganisms under different treatment times (0~24 h). As Figure 1 shows, the numbers of all bacteria after treatment with a *P. acidilactici* CCFM18 or *P. acidilactici* CCFM28 (10%, *v*/*v*) supernatant obviously decreased after 8 h, reaching their maxima at the stationary phase (16 h), after which the cell numbers remained stable. It can be concluded that *P. acidilactici* CCFM18 and *P. acidilactici* CCFM28 produced inhibitory substances beginning at 8 h, when they were in the exponential phase; then, the indicator bacteria were inhibited or killed by antimicrobial substances. The antagonistic activities were calculated as removing the *P. acidilactici* group from the control group. Of the four tested indicator bacteria, *L. thermophilus* was the most sensitive to the supernatants of the studied *P. acidilactici* CCFM18 and *P. acidilactici* CCFM28, with the highest antagonistic activities of 1.66 log CFU/mL and 2.42 log CFU/mL, respectively. This was followed by *L. delbrueckii*, with antagonistic activities of 1.36 log CFU/mL and 2.02 log CFU/mL, respectively, and *L. helveticus*, with antagonistic activities of 1.06 log CFU/mL and 1.52 log CFU/mL, respectively. Both *P. acidilactici* CCFM18 and *P. acidilactici* CCFM28 displayed relatively low antimicrobial activities against *E. faecalis*, with antagonistic activities of 0.74 log CFU/mL and 1.55 log CFU/mL, respectively. These results indicate that the supernatant of *P. acidilactici* CCFM18 or *P. acidilactici* CCFM28 inhibited the growth of four indicator bacteria in comparison with the control group, and *P. acidilactici* CCFM28 exhibited higher antagonistic activity than *P. acidilactici* CCFM18.

The antimicrobial activities of LAB were also evaluated via agar well diffusion assay. The lactic acid bacterium strains *P. acidilactici* CCFM18 and *P. acidilactici* CCFM28 have different antimicrobial activities, as shown in Table 1. Both of the LAB strain supernatants effectively inhibited the growth of the indicator strains to various degrees (the diameters of the inhibition zones varied between 7.24 ± 1.28 mm and 22.89 ± 2.82 mm). *P. acidilactici* CCFM18 and *P. acidilactici* CCFM28 showed the highest antimicrobial activity against *L. thermophilus*, with inhibition zone diameters of 22.89 ± 2.82 mm and 20.23 ± 2.03 mm, respectively. They also showed obvious antimicrobial activities against *L. delbrueckii*, with inhibition zone diameters of 16.03 ± 2.24 mm and 18.89 ± 2.37 mm, respectively, and *L. helveticus*, with inhibition zone diameters of 14.14 ± 1.87 mm and 18.03 ± 2.24 mm, respectively. *E. faecalis* was relatively less inhibited by *P. acidilactici* CCFM18 and *P. acidilactici* CCFM28, with inhibition zone diameters of 7.24 ± 1.28 mm and 10.36 ± 0.63 mm, respectively.

The antimicrobial activity of LAB has also been documented by earlier researchers [4]. They found that LAB species can help regulate microbial proliferation by producing organic acids, hydrogen peroxide, and bacteriocins generated during the fermentation process [9,23]. Papagianni and Papamichael reported that some strains of *Pediococcus pentosaceus* produce antimicrobial peptides identified as a pediocin or a class IIa bacteriocin that are heat- and cold-stable peptides with inhibitory activity against several Gram-positive bacteria and pathogens [24]. The results above show that the antibacterial effects of *P. acidilactici* CCFM18 and *P. acidilactici* CCFM28 are dependent on the LAB strain and on the indicator microorganism species. The tested LAB strains demonstrated good inhibition properties against all tested microorganisms and could be used to reduce biological contamination.

### 3.2. Effects of P. acidilactici on the Antioxidant Systems of Indicator Bacteria

**Figure 2:**
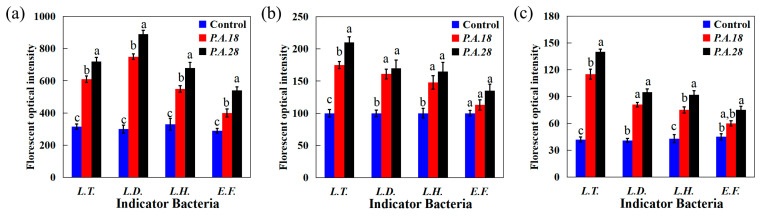
Changes in intracellular (**a**) ROS level, (**b**) SOD activity, and (**c**) MDA content in *L. thermophilus* (*L.T.*), *L. delbrueckii* (*L.D.*), *L. helveticus* (*L.H.*), and *E. faecalis* (*E.F.*) systems after 24 h of treatment with supernatants from *P. acidilactici* CCFM18 (*P.A.*18) and *P. acidilactici* CCFM28 *(P.A.*28). Different letters within the same bacteria indicate statistical difference (*p* < 0.05).

To further assess how the metabolites of *P. acidilactici* CCFM18 and *P. acidilactici* CCFM28 contributed to their antimicrobial activity, this study tested the intracellular ROS levels of the indicator bacteria. The ROS level is an important signal for both the normal physiological function of cells and antimicrobial substances that cause cellular damage [25]. As shown in Figure 2a, the intracellular ROS levels of the indicator bacteria treated with extrametabolites of *P. acidilactici* CCFM18 and *P. acidilactici* CCFM28 for 24 h were significantly higher than those of bacteria without extrametabolite treatment, indicating a state of elevated cellular oxidative stress in the presence of extrametabolites. The intracellular ROS levels of indicator bacteria incubated with metabolites of *P. acidilactici* CCFM18 increased by 0.94- (*L. thermophilus*), 1.50- (*L. delbrueckii*), 0.67- (*L. helveticus*), and 0.38 (*E. faecalis*)-fold after 24 h; these are slightly lower than the levels of increase observed for *P. acidilactici* CCFM28. ROS can cause cell death by damaging a number of cellular targets, such as the cell membrane and cell organelles or nucleic acids, proteins, and lipids [26]. The results presented above suggest that antimicrobial substances from *P. acidilactici* CCFM18 and *P. acidilactici* CCFM28 could influence indicator cells via intracellular ROS accumulation.

SOD acts as a scavenger of ROS to help cells cope with oxidative stress. The variations in SOD activity were analyzed to evaluate the degree of cellular oxidative damage [27]. The results in Figure 2b demonstrate that SOD activity increased in the indicator bacteria during 24 h of treatment with *P. acidilactici* CCFM18 and *P. acidilactici* CCFM28 extrametabolites. This suggests that SOD activity increased during the treatment process due to the oxidation stresses caused by the *P. acidilactici* CCFM18 and *P. acidilactici* CCFM28 metabolites; however, the different bacterial cells adapted to the adverse environmental stresses with different protection abilities, resulting in SOD activity of the following order in the different bacteria: *L. thermophilus > L. delbrueckii* > *L. helveticus* > *E. faecalis*. It was found that the SOD level in the *P. acidilactici* CCFM28 systems were relatively higher than those in the *P. acidilactici* CCFM18 systems, confirming that the *P. acidilactici* CCFM28 metabolites produced slightly higher oxidation stress than the *P. acidilactici* CCFM18 metabolites [28].

MDA, as a decomposition product of cell membrane lipid peroxidation, has been widely applied as a biomarker for cellular oxidative destruction [26,28]. Figure 2c demonstrates that the MDA content in both of the *P. acidilactici* CCFM18 and *P. acidilactici* CCFM28 systems increased over 24 h of incubation. This supports the results obtained regarding ROS and SOD activity in Figure 2a,b. The levels of MDA in the *P. acidilactici* CCFM28 system were also slightly higher than those in the *P. acidilactici* CCFM18 system. These results show that the levels of cell membrane permeability of the different indicator bacteria held the following order given the same treatment time: *L. thermophilus > L. delbrueckii* > *L. helveticus* > *E. faecalis*. This is in accordance with the order obtained for SOD.

According to the above findings, the antimicrobial stressors of *P. acidilactici* CCFM18 and *P. acidilactici* CCFM28 caused an increase in ROS production in the indicator cells during the treatment period. SOD is the first line of defense against ROS, and SOD activity was therefore induced in response to oxidative stress, resulting in increases in the MDA content and cell membrane permeability. Some of the intracellular ROS was scavenged by antioxidant enzymes, but eventually, the oxidative stress was too much for the enzymes in the bacteria to counteract. This resulted in the leakage of intracellular substances, including ROS, decreasing the intracellular ROS level [29]. Therefore, the MDA content of *L. thermophilus* bacteria was higher than that of the other bacteria, and its ROS level was lower than that of *L. delbrueckii* cells. The results also indicate that the oxidative stress caused by *P. acidilactici* CCFM18 was widely dispersed, and the antioxidative properties depended on the indicator strain. According to published studies, class II bacteriocin peptides are able to permeate target cell membranes and enhance ROS penetration into bacteria [30].

The composition and structure of both the cell wall and cellular membrane(s) may be the reason for the antimicrobial metabolites leading to different ROS levels in these indicator bacteria. The presence of proteases in and near the target cell may also reduce the effectiveness of antimicrobial metabolites in some cases. Finally, the unique physiological states of the *L. thermophilus*, *L. delbrueckii*, *L. helveticus*, and *E. faecalis* may affect the ease with which bacteriocin can actually form pores on the cell membrane.

### 3.3. Divergent Metabolic and Interspecies Interaction Profiles of P. acidilactici

**Figure 3:**
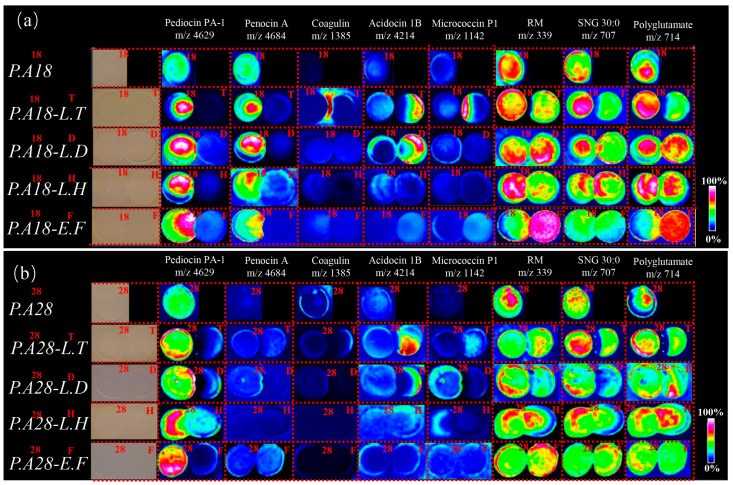
MALDI-TOF MSI analysis of (**a**) *P. acidilactici* CCFM18 (*P.A.18*) and (**b**) *P. acidilactici* CCFM28 (*P.A.28*) in monoculture and in interaction with *L. thermophilus* (*L.T.*), *L. delbrueckii* (*L.D.*), *L. helveticus* (*L.H.*), and *E. faecalis* (*E.F.*) after 48 h of co-culturing. The *m*/*z* distributions of *P. acidilactici* molecules are displayed as false color overlays of an optical image, ribonucleotide monophosphate (RM), and sulfonioglycerolipid 30:0 (SNG 30:0).

The interactions between *P. acidilactici* CCFM18 or *P. acidilactici* CCFM28 and indicator bacteria were investigated by culturing the bacteria side-by-side on MRS agar surfaces in spots with high cell density; this method has been previously applied to study microbial interactions [14]. Each of the bacteria formed distinct reaction zones near the *P. acidilactici* CCFM18 or *P. acidilactici* CCFM28 colony, referred to hereafter as the ‘interaction zone’ (Figure 3a). MSI is a label-free imaging method that has been applied to assess the spatial patterns of chemical output and metabolic exchange that occur between microbes [31]. Recently, MALDI-TOF MSI has provided a new way to examine the exchange of secondary metabolites between interacting bacteria in situ, thus identifying a novel group of peptide antibiotics that act as antimicrobial agents [31]. To gain insight into the chemical mediators of the interactions between *P. acidilactici* CCFM18 or *P. acidilactici* CCFM28 and indicator bacteria, the spatial distributions of metabolites in sections of the MRS agar surfaces containing interaction zones between *P. acidilactici* CCFM18 or *P. acidilactici* CCFM28 and indicator bacterium colonies were analyzed via MALDI-TOF MSI [13,14].

The distributions of *m*/*z* signals of the *P. acidilactici* CCFM18 and the *P. acidilactici* CCFM28 monocultures were evaluated and revealed distinct metabolic profiles (Figure 3b). Regarding bacteriocins, *m*/*z* signals corresponding to the antimicrobial pediocin PA-1 (*m*/*z* 4629) were present in both the *P. acidilactici* CCFM18 and *P. acidilactici* CCFM28 colonies; this signal was previously identified from *P. acidilactici* using MALDI-TOF MS [32,33]. However, the signal corresponding to penocin A (*m*/*z* 4684) was solely observed in the *P. acidilactici* CCFM18 colony. Recently, studies on bacteriocin producers have suggested that it may be a common phenomenon of LAB to produce more than one bacteriocin. For instance, *E. faecium* CTC492 produces not only the class IIa bacteriocin enterocin A but also produces enterocin B, which does not belong to class IIa [34]. In this study, *P. acidilactici* CCFM18 produced at least one bacteriocin, as determined via MSI. The metabolite distributions in all microbial interactions are shown in Figure 3, representing different interactions of the spatial chemical response of *P. acidilactici* CCFM18 or *P. acidilactici* CCFM28. The increased intensity of different molecules was most pronounced after 48 h at the *P. acidilactici*—*L. thermophilus* interface (Figure 3). This result indicates that antagonist interactions simulate LAB to promote the production of bacteriocin and other natural active products. The MSI analysis showed that the intensity of pediocin PA-1 in the *P. acidilactici* CCFM28—*L. thermophilus* interface was higher than that in the *P. acidilactici* CCFM18—*L. thermophilus* interface. Intriguingly, some interactions could not trigger the production of new compounds at the interaction zone with the exception of the *P. acidilactici* CCFM18—*L. thermophilus* interaction, and several of its stimulated compounds were unique. These results indicate that the chemical responses of *P. acidilactici* CCFM18 and *P. acidilactici* CCFM28 are highly idiosyncratic, depending on the interacting indicator strains. Notably, the coagulin (*m*/*z* 1385) molecule was solely present at the interface of the *P. acidilactici* CCFM18—*L. thermophilus* interaction, which overlaps with the region in which indicator bacteria do not grow [35]. LAB have evolved mechanisms to control the production of antimicrobial peptides via a phenomenon called quorum sensing (QS) [36]. QS is a cell—cell communication strategy that allows for the production of small antimicrobial peptides by sensing antagonist molecular signaling or the cell intensity [9]. The QS-triggered antibiotics and toxins behavioral responses have been investigated in several LAB, including *Camobacterium piscicola*, *Lactococcus lactis*, *Lactobacillus plantarum*, and *Lactobacillus sakei* [9]. Maldonado et al. reported that the competitiveness is capable of switching on bacteriocin production in *L. plantarum* NC8 via a QS mechanism mediated by PLNC8IF [37]. However, this is the first study to report QS-mediated antibiotic production in *P. acidilactici.* The metabolite cross-talk illustrated in the MALDI neighboring images may therefore reflect a reciprocal effect of different secretion factors on bacteriocin release from *P. acidilactici*.

Several metabolites of the indicator bacteria were suppressed on the side facing *P. acidilactici* CCFM18 or *P. acidilactici* CCFM28; of note, acidocin 1B (*m*/*z* 4214) disappeared at the *P. acidilactici* CCFM18—*L. thermophilus* interaction site and only occurred on the outer edge of the nonexposed region of *L. thermophilus* (Figure 3a). The decrease in bacteriocin contents in *L. thermophilus* suggests that the interaction of *P. acidilactici* CCFM18—*L. thermophilus* suppressed the expression of bacteriocins in the *L. thermophilus* colony. A surprising result from the MSI was that micrococcin P1 (*m*/*z* 1142)—a reported QS signaling molecule—appeared only at the *P. acidilactici* CCFM18—*L. thermophilus* interaction site. Historically, many thiazolyl peptides have been isolated from common LAB, such as *Lactobacillus gasseri* [38]. It is possible that, similar to other classes of microbial metabolites, thiazolyl peptides may act as signaling cues in the interaction between *P. acidilactici* CCFM18 and *L. thermophilus*. This result also demonstrates that thiazolyl peptides cannot prevent the invasion of *P. acidilactici* CCFM18 but can sense the inhibition of nearby colonies and consequently enhance their toxicity. Ions at *m*/*z* 339 (ribonucleotide monophosphate), *m*/*z* 707 (sulfonioglycerolipid 30:0), and *m*/*z* 714 (partially characterized polyglutamate) were observed for all strains, and their distribution confirmed the inhibitory effect of *P. acidilactici* on the indicator strains. In particular, polyglutamate (*m*/*z* 714) was identified as a component of the cell wall material. The distribution of these polyglutamates confirmed the effect of *P. acidilactici* on the cell wall of the indicator bacteria, as mentioned above.

These results suggest that the production of diverse bacteriocins induces a more complex QS-mediated inhibition of the indicator bacteria by *P. acidilactici* CCFM18 than by *P. acidilactici* CCFM28. The stronger antimicrobial activity of *P. acidilactici* CCFM28 could be attributed to its growth or the fact that it produces more pediocin PA-1. However, because bacteriocins produced by LAB usually have a narrow inhibitory spectrum and are only active on closely related bacteria, the various bacteriocins produced by *P. acidilactici* CCFM18 offer promising application potential.

### 3.4. Identification of Putative Bacteriocin Gene Clusters

**Figure 4:**
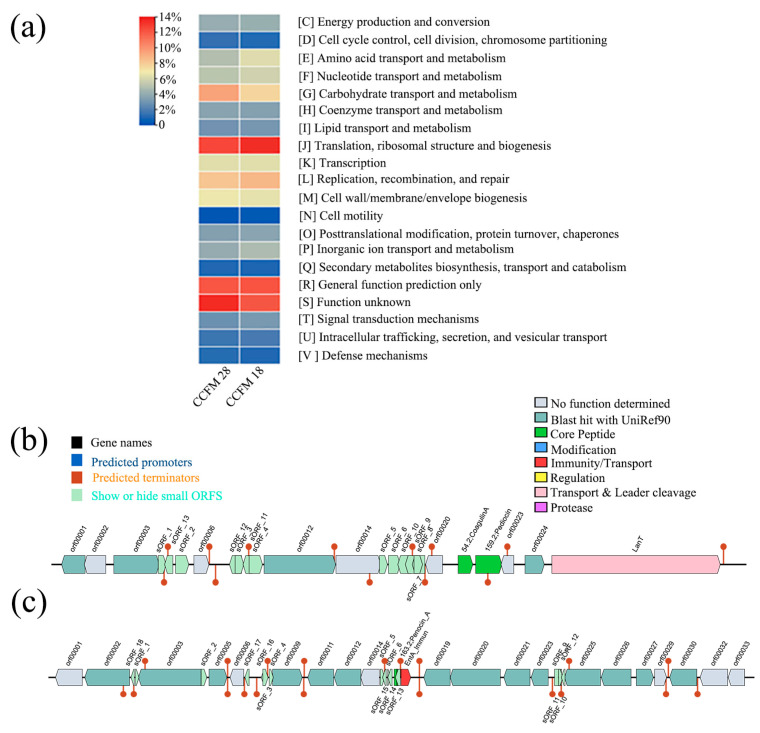
Contig of *P. acidilactici* CCFM18 and *P. acidilactici* CCFM28 (**a**), and gene clusters of *P. acidilactici* CCFM18 belonging to Operon 1 (**b**) and Operon 2 (**c**).

Clusters of Orthologous Groups (COGs) are commonly used for the functional annotation of novel genomes and various genome-wide evolutionary assessments [21]. To check whether the *P. acidilactici* CCFM18 strain and *P. acidilactici* CCFM28 strains could be from different genera, the COG database was applied to reannotate the genomes of *P. acidilactici* CCFM18 and *P. acidilactici* CCFM28. The metabolism function annotation analysis of these two strains was selected from all annotations, and displayed in Figure 4. The results indicate that *P. acidilactici* CCFM28 contains more carbohydrate transport and metabolism COGs and functional unknown COGs, whereas *P. acidilactici* CCFM18 contains more amino-acid transport and metabolism COGs and translation, ribosomal structure, and biogenesis COGs. However, *P. acidilactici* CCFM18 and *P. acidilactici* CCFM28 have a similar number of defense mechanism COGs, including bacteriocin COGs. Thus, the COG results confirmed that *P. acidilactici* CCFM18 and *P. acidilactici* CCFM28 are of distinctly different genera, but the difference between the genera in terms of bacteriocin coding needs to be further analyzed.

The detection of bacteriocin sequences in newly sequenced genomes is still a challenge. Short sequences are poorly analyzed by the Basic Local Alignment Search Tool (BLAST), and similar sequence detection methods and the analysis of uncurated small ORFs may lead to the annotation of many false small ORFs [22]. This is particularly the case for bacteriocins because they are a very varied group of antimicrobial peptides generated by bacteria and are generally encoded by small, poorly conserved ORFs [39]. BAGEL makes ORFs independent of GenBank annotations and thus avoids the omission of small non-conserved ORFs, which are the most frequent candidates for bacteriocin genes [22]. BAGEL has been successfully applied to reveal putative bacteriocin genes in different species of LAB, such as *L. lactis* IL1403, *L. plantarum*, and *Streptococcus pneumoniae* R6 [22]. In this study, BAGEL 4 was utilized for genome mining of bacteriocins and the biosynthetic clusters of *P. acidilactici* CCFM18. The data confirmed that certain gene clusters that encode the class II bacteriocins pediocin PA-1, penocin A, and coagulin are present in the genome of *P. acidilactici* CCFM18 (Figure 4). Research conducted by Martínez et al. confirmed that the biosynthesis gene-coded pediocin PA-1 is present on the chromosome of *P. acidilactici* CCFM28 [40]. The search for a new LAB with a wide spectrum of antimicrobial activities is of outstanding importance for applications in the environment, agriculture, and the food industries. Genome mining analysis of *P. acidilactici* CCFM18 confirmed that it may be a successful candidate for bacteriocin production.

## 4. Conclusions

We have demonstrated that the novel isolated LAB *P. acidilactici* CCFM18 induces different bacteriocin production and QS generation in its interactions with a wide range of antagonists. We used MSI to rapidly identify potential mediators of these diffusible interactions between *P. acidilactici* CCFM18 and its antagonists. A molecular genetics analysis identified putative biosynthetic clusters responsible for class II bacteriocin, pediocin PA-1, penocin A, and coagulin production.

We provide the first evidence that *P. acidilactici* CCFM18 can induce and invade specialized LAB or pathogenic bacteria by generating different bacteriocins. QS mediated the inhibition effect of *P. acidilactici* CCFM18 against different indicator bacteria. As the bacteriocins produced by LAB usually have a narrow inhibition spectrum and are active only on closely related bacteria, the production of various bacteriocins by *P. acidilactici* CCFM18 may have significant implications for the environmental persistence of pathogens in food. The results suggest that excessive ROS production by *P. acidilactici* CCFM18 led to the oxidation of bacterial proteins and destroyed the functional chaperones, leading to instability of the cell membrane. Further research in our lab aims to extract the individual bacteriocins generated by *P. acidilactici* CCFM18 and determine their antimicrobial effects.

## Figures and Tables

**Table 1:** Diameters (mm) of inhibition zones generated by supernatants of studied lactic acid bacteria (LAB) against selected indicator microorganisms.

LAB	Zone of Inhibition/mm			
	*L. delbrueckii*	*L. reuteri*	*L. helveticus*	*E. faecalis*
*P. acidilactici* CCFM18	22.89 ± 2.82	16.03 ± 2.24	14.14 ± 1.87	7.24 ± 1.28
*P. acidilactici* CCFM28	20.23 ± 2.03	18.89 ± 2.37	18.03 ± 2.24	10.36 ± 0.63

All experiments were performed in triplicate and expressed as the mean ± standard deviation.

## Data Availability

The original contributions presented in the study are included in the article, further inquiries can be directed to the corresponding author.
